# Effects of dorsolateral prefrontal cortex lesion on motor habit and performance assessed with manual grasping and control of force in macaque monkeys

**DOI:** 10.1007/s00429-016-1268-z

**Published:** 2016-07-09

**Authors:** S. Badoud, S. Borgognon, J. Cottet, P. Chatagny, V. Moret, M. Fregosi, M. Kaeser, E. Fortis, E. Schmidlin, J. Bloch, J. F. Brunet, E. M. Rouiller

**Affiliations:** 1 Laboratory for sensorimotor and Multisensory Integration, Research Cluster Neurosciences, Department of Medicine, Fribourg Cognition Center, University of Fribourg, Chemin du Musée 5, 1700 Fribourg, Switzerland; 2 Department of Neurosurgery, Lausanne University Hospital (CHUV), Lausanne, Switzerland

**Keywords:** Dorsolateral prefrontal cortex (dlPFC), Manual dexterity, Grip force control, Motor habits, Precision grip

## Abstract

In the context of an autologous adult neural cell ecosystem (ANCE) transplantation study, four intact adult female macaque monkeys underwent a unilateral biopsy of the dorsolateral prefrontal cortex (dlPFC) to provide the cellular material needed to obtain the ANCE. Monkeys were previously trained to perform quantitative motor (manual dexterity) tasks, namely, the “modified-Brinkman board” task and the “reach and grasp drawer” task. The aim of the present study was to extend preliminary data on the role of the prefrontal cortex in motor habit and test the hypothesis that dlPFC contributes to predict the grip force required when a precise level of force to be generated is known beforehand. As expected for a small dlPFC biopsy, neither the motor performance (score) nor the spatiotemporal motor sequences were affected in the “modified-Brinkman board” task, whereas significant changes (mainly decreases) in the maximal grip force (force applied on the drawer knob) were observed in the “reach and grasp drawer” task. The present data in the macaque monkey related to the prediction of grip force are well in line with the previous fMRI data reported for human subjects. Moreover, the ANCE transplantation strategy (in the case of stroke or Parkinson’s disease) based on biopsy in dlPFC does not generate unwanted motor consequences, at least as far as motor habit and motor performance are concerned in the context of a sequential grasping a small objects, which does not require the development of significant force levels.

**Electronic supplementary material:**

The online version of this article (doi:10.1007/s00429-016-1268-z) contains supplementary material, which is available to authorized users.

## Introduction

During the last decades, the dorsolateral prefrontal cortex (dlPFC) has been extensively studied, revealing its role in the integration of multiple cognitive attributes in the context of working memory, as well as its implication in risk related decision making (e.g., Goldman-Rakic 1987; Petrides 1994; Petrides and Pandya 1999; Watanabe and Sakagami 2007; Barber et al. 2013). Several investigations conducted on non-human primates also emphasized an implication of dlPFC in the mental representation of spatiotemporal motor sequences, where the subjects had to reproduce a sequence of actions after a delay (Barone and Joseph 1989; Pochon et al. 2001; Ninokura et al. 2004; Shima et al. 2007; Berdyyeva and Olson 2010). Still in relation to motor control, but in humans, evidence was provided for a role of dlPFC, together with basal ganglia (global neural circuit), in the control and prediction of grip force contributing to manual dexterity (e.g., Ehrsson et al. 2000, 2001; Vaillancourt et al. 2007; Wasson et al. 2010; Neely et al. 2013), complementing the expected major grip-related activities in the sensorimotor cortex (M1/S1), the premotor cortex, the supplementary motor area, the cingulate motor area, the posterior and inferior parietal cortex and cerebellum (Ehrsson et al. 2000, 2001; Kinoshita et al. 2000; Muley et al. 2001; Kuhtz-Buschbeck et al. 2008, 2001). As pointed out by Ehrsson et al. (2000), such dlPFC activity found in relation to precision grip may also reflects parallel behavioral factors, such as spatial attention, short-term memory of tactile information, selection of motor response, and attentive auto-monitoring of motor performance. However, while its role in motor learning is well established, dlPFC’s activation seems to progressively vanish when a motor task becomes more and more “automatic”, possibly reflecting delegation of responsibility to “lower” brain structures (Eliassen et al. 2001; Halsband and Lange 2006).

More recently, Kaeser and colleagues reported original data underlying the role of dlPFC in motor habit representation (Kaeser et al. 2013). In this study, the authors performed cortical biopsies in dlPFC on two macaque monkeys (*Macaca fascicularis*) and assessed their impact on sequential motor behavior (habit). More specifically, the “modified Brinkman board” task was used to quantify “free-will” spatiotemporal retrieval of pellets, performed with precision grip movements executed unimanually (Brinkman and Kuypers 1973; Liu and Rouiller 1999; Schmidlin et al. 2011; Kaeser et al. 2013). In comparison with control monkeys, dlPFC (area 46) lesioned animals exhibited a significant impact on the spatiotemporal sequences (order to visit the wells), whereas the motor performance per se (score) remained unaffected (Kaeser et al. 2013). Moreover, there was a first indication of a relationship between the size of the dlPFC biopsies and the extent of motor habit changes, as a small biopsy impacted less on motor sequences than a larger biopsy (Kaeser et al. 2013). Nevertheless, due to their limited number of cases (*n* = 2), clearly, more data are required to support this hypothesis, both in terms of number of cases as well as variability in precise location of dlPFC lesions. In addition, functional magnetic resonance imaging (fMRI) investigations conducted on human subjects also emphasized a role of dlPFC in the execution of motor tasks requiring some control (prediction) of the grip force to be exerted (Wasson et al. 2010).

The present report corresponds to the initial step of a broader study aiming at testing a novel therapeutic strategy based on autologous adult neural cell ecosystem (ANCE) transplantation (e.g., Brunet et al. 2005; Kaeser et al. 2011; Bloch et al. 2014) in a non-human primate 1-methyl-4-phenyl-1,2,3,6-tetrahydropyridine (MPTP) model of Parkinson’s disease. In this context, unilateral biopsies in dlPFC were performed in four intact adult female macaque monkeys (several months before MPTP treatment) to provide the cellular material needed to obtain the ANCE. The monkeys were previously trained to perform quantitative motor (manual dexterity) tasks, including the “modified Brinkman board” task and the “reach and grasp drawer” task (see Schmidlin et al. 2011; Kaeser et al. 2014). The aim of the present study on non-human primates was thus to extend preliminary data on the role of the prefrontal cortex (PFC) in motor habit and predominantly test the hypothesis that dlPFC indeed contributes to predict the grip force required when a precise level of force to be generated is known beforehand, as recently reported in humans (Wasson et al. 2010).

## Materials and methods

### Subjects

The data were collected from a group of four adult female macaque monkeys (*Macaca fascicularis*) weighting from 3.0 5.0 kg (Mk-MY, Mk-LY, Mk-MI, and Mk-LL) and aged between 4 and 8 years old at the beginning of the behavioral training, which begun up to 3 years before the present data collections. In other words, the monkeys were highly trained for the two motor tasks considered here (see below). The four monkeys were housed together in a 45 m^3^ room, in which they were free to move and interact with each other. In addition, the room was equipped with different enrichment features, including an outdoor space and free access to water (see www.unifr.ch/spccr/about/housing). Each monkey worked every day with an experimenter on one or two different behavioral tasks. Before being transferred to the behavioral laboratory, each animal was first transferred in a free-will manner into a primate chair and was weighted to monitor its welfare. In addition, the appetite, the social behavior and the fur state were controlled daily during the entire experiment. After performing the behavioral tests, the monkey received its daily ration of food composed of complete primate cereal croquettes, vegetables, and fruits. All surgical and behavioral procedures were approved by the local ethical committee in accordance with the guidelines for the Care and Use of Laboratory Animals and approved by local (Canton of Fribourg) and federal (Swiss) veterinary authorities (authorization numbers 22010, 17/09, and 18/10).

### Behavioral tasks

Manual dexterity assessment was based first on the “modified-Brinkman board” task (adapted from the original task of (Brinkman and Kuypers 1973), which consisted of pellets retrieval from 25 horizontal and 25 vertical wells, randomly distributed in a Plexiglas board, each well containing a banana-flavored food pellet (Rouiller et al. 1998; Schmidlin et al. 2011). The size and the shape of the wells forced the monkey to use the precision grip (opposition of the thumb and the index finger) to successfully retrieve the food pellets. The task was performed for each hand separately, 3 days a week. The number of pellets correctly retrieved within the first 30 s corresponded to the score, reflecting the motor performance (in Mk-MY, Mk-LY, and Mk-MI). The motor performance in Mk-LL was assessed in a different manner. Indeed, Mk-LL adopted a mix of two behaviors, either grasping one pellet after the other as expected or by sometimes retrieving several pellets in a row to store them into the hand palm before bringing all of them to the mouth, as illustrated in Kaeser et al. (2014). Due to such random variation, MK-LL motor performance was thus calculated by summing the total number of single pellets correctly retrieved and the multiple pellets correctly retrieved during the entire task, corresponding to the “total score”.

In addition, the motor strategy (habit) was assessed based on the temporal picking sequence (order to visit the 50 wells one after the other). However, the motor strategy given by the sequential order to visit the wells remains a qualitative assessment of the motor habit. To quantify the motor habit data, the same statistical approach as used by (Kaeser et al. 2013) was applied. Each well received a spatial position number according to its position along the horizontal left–right axes of the “modified-Brinkman board” (a left located well received a small number, whereas a right located well was associated to a large number; range 0–50 corresponding to the total number of wells). The spatial position number of each well was then subtracted from the order number in the temporal sequence. The absolute values of the 50 differences were summed up giving an index of systematic motor sequence. For instance, when a monkey performed the “modified-Brinkman board” task from the leftmost wells moving progressively to the rightmost zone of the board along the horizontal axis, the index of motor habit is a small number, as the difference between spatial location (left = small number) and temporal sequence is small. In contrast, a systematic scan of the board from right to left yields a large index of motor habit, as for each well, the difference between spatial location number and sequential order number is large. This index permitted to assess whether the monkey repeated the same sequence along the daily sessions or not (Fig. 2d; Supplementary Fig. 3). For instance, a great variability in this index reflects changes in the picking sequence from one daily session to the next, whereas a small variability reflects stable picking sequence along the consecutive daily sessions. Note that the motor strategy of Mk-LL could not be assessed as the monkey did not perform the “modified Brinkman board” task following the standard individual pellet grasping procedure (see above).

The second motor task was the “reach and grasp drawer” task, used to quantify the production of controlled grip and load (pull) forces, as well as their time course (see Schmidlin et al. 2011; Kaeser et al. 2013). This task was designed, so that the monkey had to pull open a drawer against different resistances, using one hand at the time (as derived from previous versions: (Kazennikov et al. 1994, 1999; Kermadi et al. 1997, 1998). The “reach and grasp drawer” task required holding firmly the drawer knob between the thumb and index finger (grip force), as well as exerting a force to pull the drawer (load force), which were both monitored. One standard session consisted of ten correct consecutive trials at each different resistances (R0 = 0 Newton, R3 = 1.25 Newton and R5 = 2.75 Newton), performed with each hand. A correct trial was defined as successful drawer opening followed by adequate pellet retrieval using precision grip (opposition of the thumb and index finger). Each session started with the smallest resistance (R0) corresponding to almost no resistance. Once ten correct trials were performed at R0, the monkey received an extra reward (a piece of almond) and the resistance was then raised to R3. After ten correct trials at R3, again extra-rewarded, the resistance was increased to R5. Once the three resistances have been performed with one hand, the same paradigm was followed for the other hand. Two different parameters were analyzed in the present report. The first one was the maximal grip force developed in each trial. The second one was the maximal load force, also measured in each trial. The first trial at each resistance was removed from the main analysis, as it represents an outlier (unknown resistance at the onset of a new series of trials). In a separate analysis, the forces produced at the first trials at each resistance were compared with those at subsequent trials at the same resistance. The four monkeys performed this task two-to-three times a week.

One of the monkeys (Mk-MI) performed the drawer task correctly with the left hand only (due to an injury of the right hand). Indeed, Mk-MI did not use a precision grip movement to hold the drawer’s knob with its right hand, but used an alternative strategy (single finger push on the upper side of the knob), preventing any measurement of grip force. Despite this, Mk-MI performed the “modified Brinkman board” task correctly with both hands.

### Surgical procedure (cortical biopsy)

**Fig. 1:**
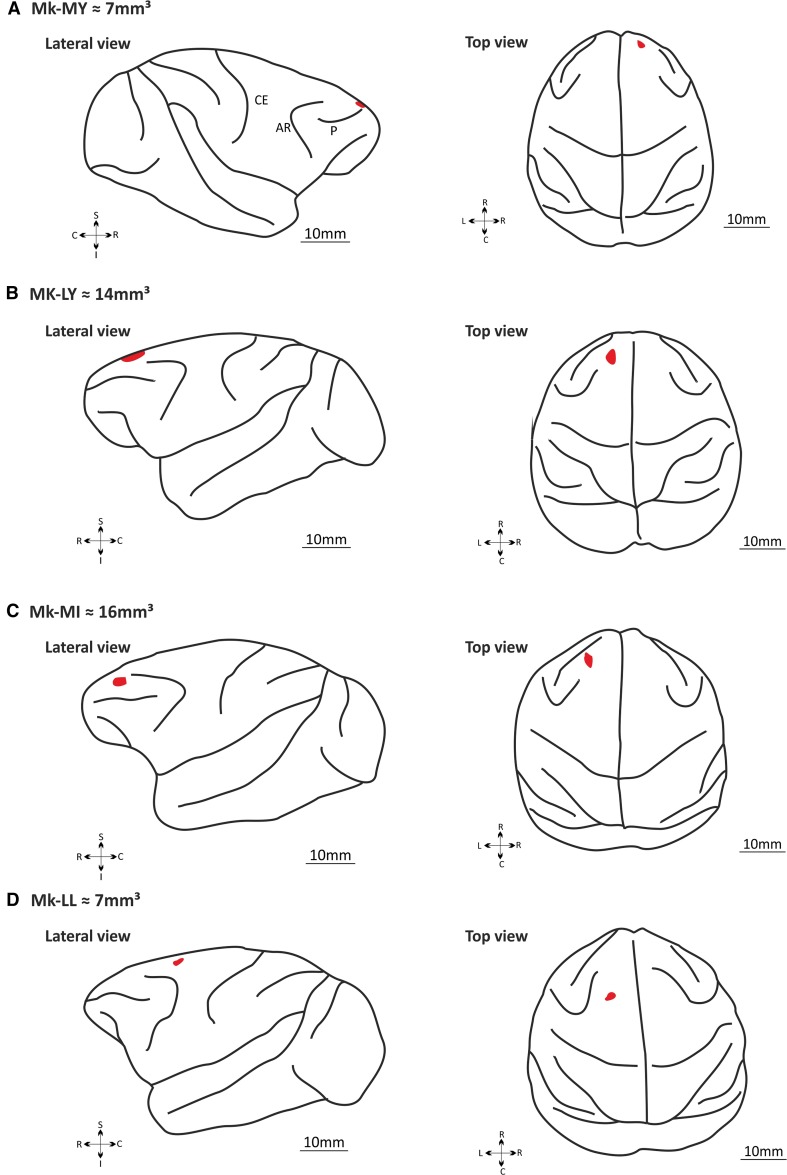
On schematic representations of the brain of the four monkeys, the dLPFC/PMd-r biopsies’ location and extent are represented by a red spot. Each biopsy’s volume (*gray* matter) and position was estimated from MRI images using the software FSLView v3.2.0. **a** represents the lateral (*left*) and the top view (*right*) of Mk-MY’s brain with the biopsy in *red* (volume = 7 mm^3^). **b** represents the lateral (*left*) and the top view (*right*) of Mk-LY’s brain with the biopsy in red (volume = 14 mm^3^). **c** represents the lateral (*left*) and the top view (*right*) of Mk-MI’s brain with the biopsy in red (volume = 16 mm^3^). **d** represents the lateral (*left*) and the top view (*right*) of Mk-LL’s brain with the biopsy in red (volume = 7 mm^3^). Legends: lateral view of the brain: *S* superior (medial), *I* inferior (lateral), *R* rostral, *C* caudal; top view of the brain: *R* rostral, *C* caudal, *L* left, *R* right. *CE* central sulcus, *AR* arcuate sulcus; *P* principal sulcus

Before surgery, each animal was first lightly sedated under ketamine (Ketasol^®^, 10 mg/kg), midazolam (Dormicum^®^, 0.1 mg/kg) and methadone (0.2 mg/kg), and prepared for the surgery. In addition, each animal received an intramuscular dose of methadone (Methadon^®^; Streuli; 0.2 mg/kg) and was treated with analgesic Carprofen (Rymadil^®^; Pfizer; 4 mg/kg; subcutaneously), atropine (atropine; 0.05 mg/kg; intramuscularly) to reduce bronchial secretions, antibiotics (Synulox^®^; Pfizer; 8.75 mg/kg; subcutaneously), and dexamethasone (Dexadreson^®^; Intervet; 0.3 ml/kg; diluated 1:1 in saline; intramuscularly). Once the animal was in the surgery room, it was put under intravenous (femoral vein) perfusion with 1 % propofol (Frescenius^®^) diluted with ringer lactate solution and 125 mg of ketamine hydrochloride (20 ml of propofol for 40 ml of Ringer lactate and 1.25 ml of ketamine) to ensure deep anesthesia. The infusion rate was modulated to maintain an optimal level of anesthesia. During the entire surgical procedure, the level of anesthesia and the physiological state were controlled based on the arterial oxygen saturation, heart rate (ECG), ventilation (rate and expired CO_2_), and body temperature. The animal was then placed in a stereotaxic framework to fix its head with ear bars for the surgery. To reduce possible pain resulting from the fixation points, ear bars were coated with a local analgesic cream (Lidohex^®^). Local injections of lidocaine (Rapidocain^®^) were used to anesthetize the incision site. After the incision, the muscle tissue was pushed on the side to expose the skull, allowing craniotomy above the rostral extent of the frontal lobe (aimed to dlPFC). However, to reduce the impact of the craniotomy, the size of bone opening was tentatively made as small as possible; as a consequence, the various sulci (e.g., arcuate and principal sulci) could not be clearly identified to guide the precise location of the biopsy, which turned out to be variable from one monkey to the next (Fig. 1). In three monkeys, the skull opening was made on the left side (Mk-LY, Mk-MI, and Mk-LL), whereas it was on the right side in Mk-MY. The size of the bone flap was about 1 cm^2^. After bone removal, the dura mater was incised and a piece of dlPFC cortical tissue was removed and directly placed into storage medium. The injured blood vessel was cauterized, the bone flap put back in place and fixed with histological glue (Histoacryl^®^). The muscle tissue and the skin were sutured. After the surgery, each animal was surveyed until its total awakening. It was considered as stable when the monkey started to eat and drink again. A posology composed of Caprofen (Rymadil^®^, ½ pill twice a day) and antibiotics (Clavubactin^®^, 1 pill twice a day) was followed during ten days.

### Magnetic resonance imaging (MRI)

MRI was used to determine the precise position of the biopsies, while the monkeys were still alive, before engaged in the MPTP subsequent protocol. Each animal was first lightly sedated with ketamine (Ketasol^®^, 10 mg/kg) and midazolam (Dormicum^®^, 0.1 mg/kg). After being transported to the MRI facility (radiology, Hospital of Fribourg, Switzerland), each monkey was anesthetized via an intravenous perfusion of 1 % propofol (Frescenius^®^) diluted with ringer lactate solution and 125 mg of ketamine hydrochloride (20 ml of propofol for 20 ml ringer lactate and 1.25 ml of ketamine). The infusion rate was adjusted to ensure an optimal level of anesthesia (ECG and O_2_ saturation were continuously monitored). In addition, gloves filled with hot water were placed around the monkey’s body to maintain its body temperature. The monkey was placed in the magnet in a prone position with a flow of oxygen (3 l/min) in front of the nose. Data were acquired on a GE 3T magnet using 3D transverse T1-weighted acquisition protocol. The parameters were as follows: field of view: 256 × 256, TR: 7.248, TE: 3.032, and FS: 3. Images were then rotated because of the prone position of the animal (FSLview V3.2.0). After the proper rotation, brains were extracted from the skull and represented in a three-dimensional view before being schematized. The positions of each biopsy and their volumes (corresponding to the gray matter) were estimated based on the MRI images. Note that histological verification of the biopsy could not take place, as a second dlPFC biopsy took place in the vicinity of the first one when the MPTP treatment was ongoing. It was, therefore, not possible to distinguish the two biopsies, and only the first one is relevant for the present behavioral study, before MPTP treatment.

### Statistical analyses

Intra-individual comparisons (pre-biopsy/post-biopsy) were performed in SigmaPlot/SigmaStat (13.0). Depending on data distribution (normal or not), the statistical tests applied were either an unpaired Student *t* test or a Mann and Whitney test. Besides the intra-individual comparisons, an overall statistical analysis, including all monkeys, was conducted in the open access environment “R” (version 3.2.1) available online. The two experimental phases (i.e., pre-biopsy/post-biopsy) were compared with a linear mixed model (lme, package nlme). Load force and grip force were considered as the responses of variables, the experimental phases, resistances, and the interaction of these last two variables as fixed effects. The random effects comprised the hand (left or right), which was nested within monkeys’ identities. The significant threshold was fixed at 0.05.

## Results

### Location and size of the biopsies

Based on MRI, the extent and position of the cortical biopsies were identified and reconstructed. Transposed to the surface of the corresponding brains, the biopsies are illustrated in Fig. 1, for Mk-MY, Mk-LY, Mk-MI, and Mk-LL. The volumes of the dlPFC biopsies were 7, 14, 16, and 7 mm^3^, respectively. In Mk-MY, the biopsy is located at the most rostral part of dlPFC, about 5 mm from the midline, most likely overlapping the transition zone between Brodmann’s cortical area 9 and area 10. In addition, about 5 mm lateral with respect to the midline, but somewhat more caudal, the biopsy in Mk-LY appears to be located in the rostral part of area 9. The lesion in Mk-MI is located in a zone of dlPFC comparable to that of Mk-LY, though somewhat more lateral. In the fourth monkey (Mk-LL), the biopsy is located clearly more caudally, slightly anterior to the genu of the arcuate sulcus and at a medio-lateral level consistent with a location in the rostral dorsal premotor area (PMd-r, area F7), close to the more medial pre-supplementary motor area (SMA, area F6). In other words, Mk-LL should be treated here as an outlier, considering that its biopsy did not involve dlPFC. The four monkeys included in the present study differ from the two animals subjected to dlPFC lesion in Kaeser et al. (2013), as in the latter study, the two biopsies were located in area 46.

### Modified Brinkman board task

**Fig. 2:**
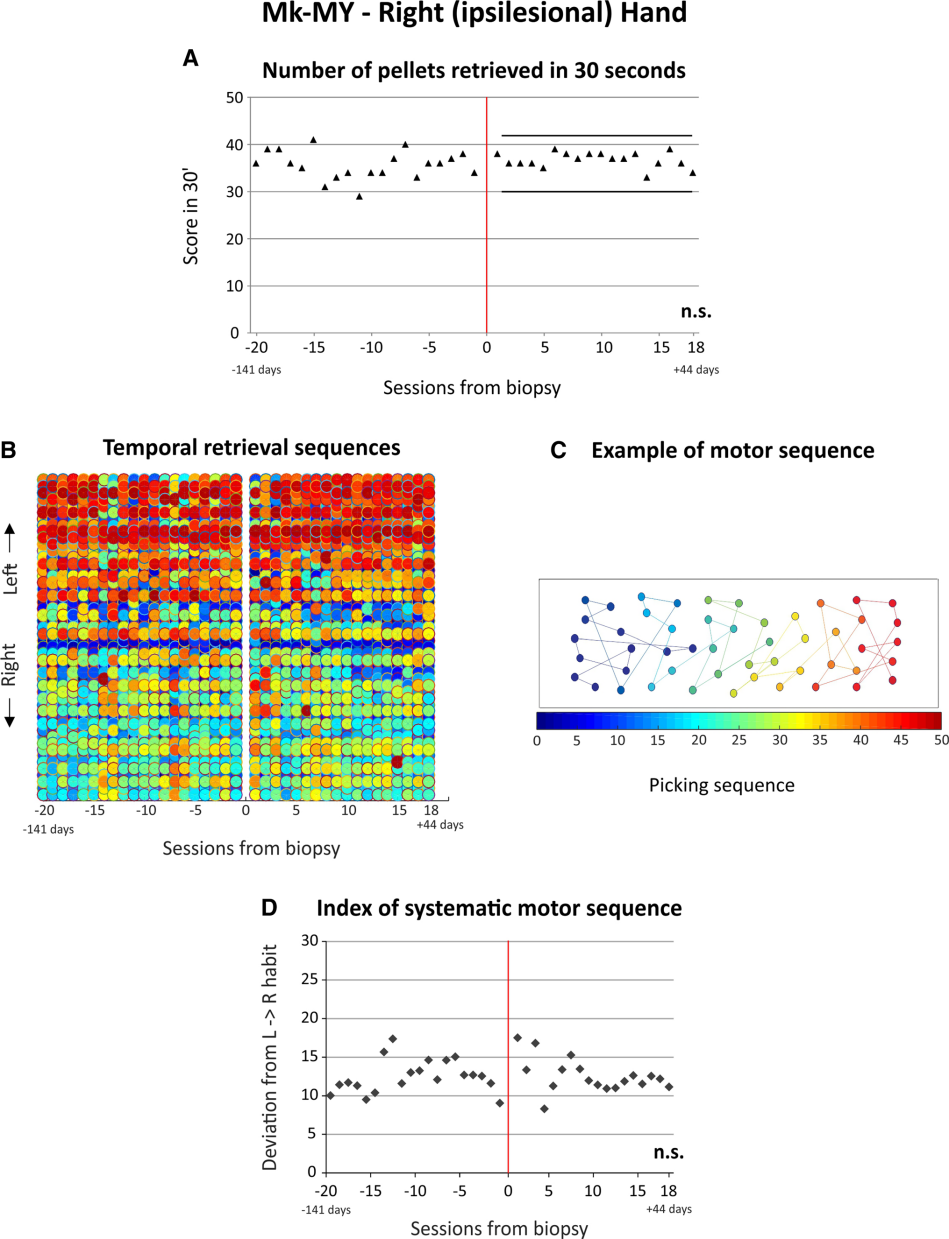
Results derived from the “modified Brinkman board” task for the ipsilesional hand (*right*) in Mk-MY. **a** represents the score that corresponds to the number of pellets retrieved during the first 30 s. The *X*-axis corresponds to the behavioral sessions (note that the time is represented as “sessions from biopsy“, because in the pre-biopsy phase, one session per week during 4 months was analyzed, as the monkeys were considered to be at a plateau of motor performance, whereas during the post-biopsy phase, all sessions were analyzed. This is also true for the picking sequence and the “reach and grasp drawer” task data). The *Y*-axis corresponds to the total numbers of pellets (*horizontal* and *vertical* wells) retrieved during the first 30 s (total score in 30′). The *red* *line* corresponds to the day at which the cortical biopsy took place. The two *black* *horizontal* *lines* indicate superior and inferior limits, defined as mean pre-biopsy value plus two standard deviations (SDs) and mean pre-lesion value −2 SDs, respectively. **b** represents the picking sequence along the *left*–*right* axes of the board. The *X*-axis represents the daily behavioral sessions, so that one column corresponds to one individual session. The *Y*-axis represents the 50 wells of the board, ordered according to the *left*–*right* axes. The *colors* correspond to the temporal picking sequence. The first pellet retrieved is represented in *dark* *blue* and the last one in *dark* *red*. The entire sequence is thus represented in a color gradient fashion, ranging from the *darkest* *blue* (first pellet retrieved) to the *darkest* *red* (last pellet retrieved). The session 0 corresponds to the time point of the cortical biopsy. **c** is a schematic representation of an individual “modified Brinkman board” session. In this example, the subject began to pick up the pellets from the *left* extremity of the board (*blue* *dots*) to progressively move towards the *right* extremity (*red* *symbols*). **d** represents the quantitative assessment of motor sequence in the “modified Brinkman board” task. The *black* *dots* represent the index of systemic motor sequence. The *X*-axis corresponds to the behavioral daily sessions starting at the time of the biopsy (0 in the abscissa). Negative days are pre-biopsy and positive days are post-biopsy. The *Y*-axis represents the extent of deviation from an “ideal” systematic motor sequence starting from the *left* of the board and finishing at the *right* *side* of the board. In other words, a picking sequence going from *left* to *right* gives a low score, whereas a picking sequence going from *right* to *left* gives a high score. The indexes were compared pre- versus post-biopsy, based on the non-parametric Mann and Whitney test (MW) or the parametric unpaired Student *t* test. The results for each statistical comparison are indicated at the bottom right of each graph: *n.s.* non-significant difference (*p* > 0.05). Comprehensive data for the “modified Brinkman board” task are shown in Supplementary Figs. 1–3

The data derived from the behavioral score, given by the number of pellets retrieved in 30 s, showed that the monkeys exhibited a largely stable manual dexterity performance before dlPFC biopsy, as illustrated for Mk-MY in Fig. 2a. The behavioral score data for both hands for all four monkeys are shown in Supplementary Fig. 1, together with the results of statistical analyses (non-parametric Mann and Whitney test or parametric unpaired Student *t* test). Similarly, the data obtained from the temporal picking sequence analysis show that the monkeys followed a largely reproducible strategy (motor habit) to empty the board before the biopsy, and in other words, the temporal sequence to visit the 50 wells, along the left–right axes (Fig. 2b, illustrated for Mk-MY; see Supplementary Figs. 2 and 3 for a comprehensive presentation of the motor sequence data). Interestingly, neither the score nor the temporal sequences to visit the wells were strongly affected by the dlPFC or PMd-r biopsy, as illustrated for Mk-MY in Fig. 2a and b. Indeed, statistical analyses comparing pre- versus post-biopsy scores and temporal sequences did not show any statistically significant difference (*p* > 0.05). These conclusions apply for both the ipsilesional and contralesional arms in the four monkeys, with, however, the exceptions of the contralesional hand in Mk-MY and the contralesional hand in Mk-LY for the score (Supplementary Fig. 1A). In these two panels (A and D), the statistically significant difference indeed showed no deficit of score post-biopsy, as the score was actually higher post-biopsy, thus supporting the absence of detrimental effect of the lesion; on the contrary, the performance was improved, possibly due to more practicing. In addition, the index of systematic motor sequence for the contralesional hand of Mk-MI showed a significant difference between the pre- and post-biopsy periods (Supplementary Fig. 3F). However, the difference is due to a change of motor sequence which was not time linked to the biopsy, as it took place ten sessions before the biopsy. At that step, we can conclude that the dlPFC biopsies, with the characteristics as performed in the present study (size and/or precise location), did not systematically impact on performance and motor habit in the “modified Brinkman board” task, in contrast to larger and differently located biopsies (area 46) performed earlier (Kaeser et al. 2013).

### Reach and grasp drawer task

**Fig. 3:**
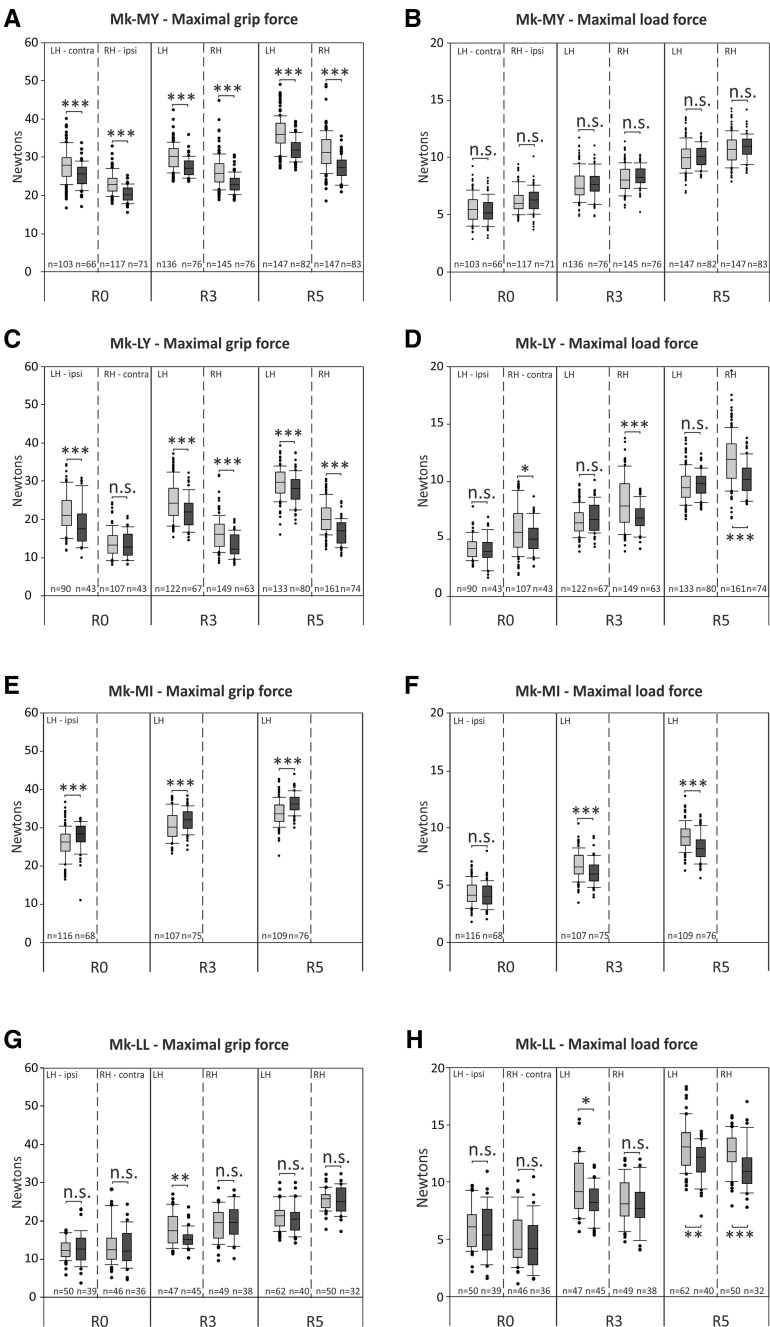
*Box* and* whiskers* graphs show the quantitative assessments in the “reach and grasp drawer” task, separately for the three resistances, namely, R0, R3, and R5. For each resistance, the *left* hand (LH) and the *right* hand (RH) are represented. *Box* *plots* are composed of all correct trials before (pre-, represented in *gray*) and after (post-, represented in *black*) the cortical biopsies. The total number of correct trials included in each *box* and *whiskers* is indicated at the bottom of each column (*n =*). Statistical analyses (parametric Student unpaired *t* test/Mann–Whitney test) compare maximal grip and load forces between pre-biopsy and post-biopsy sessions, for each resistance and for each hand. Statistically significant differences are indicated: * is for *p* ≤ 0.05, ** for *p* ≤ 0.01, *** for *p* ≤ 0.001, « n.s. » meaning statistically non-significant (*p* > 0.05). **a**, **b** show the maximal grip force and maximal load force for Mk-MY, in which the *right* hand is the ipsilesional hand. **c**, **d** show the maximal grip force and maximal load force for Mk-LY, in which the *right* hand is the contralesional hand. **e**, **f** show the maximal grip force and maximal load force for Mk-MI, in which the *left* hand is the ipsilesional hand. For Mk-MI only the *left* hand was analyzed (not able to perform precision grip with the *right* hand). **g**, **h** show the maximal grip force and maximal load force for Mk-LL, in which the *right* hand is the contralesional hand

The results obtained from the reach and grasp drawer task were separated into the three resistances opposing drawer opening, namely, R0, R3, and R5 (Fig. 3). For each resistance, the data were split into pre-biopsy and post-biopsy periods. Each box and whisker plot encompassed all correct trials for the corresponding period. For each hand, the pre- and post-biopsy periods were represented next to each other to facilitate direct comparison. The quantitative data show that the resistance had an impact on the maximal grip force and the maximal load force during both the pre-biopsy and post-biopsy periods. Indeed, and as expected, both maximal forces increased in parallel with the resistances, the higher the resistance, and the higher the force required to grasp the knob or to pull the drawer.

When comparing pre-biopsy versus post-biopsy periods (gray versus next black boxes), a statistically significant decrease of maximal grip force was observed post-biopsy for both hands in the two animals subjected to dlPFC biopsy in areas 9/10 (Mk-MY and Mk-LY), with the exception of the right (contralesional) hand of Mk-LY at resistance R0 (Fig. 3a, c). Mk-MI, subjected to dlPFC biopsy in the same rostro-caudal position than Mk-LY but slightly more lateral (area 9), exhibited a statistically significant increase of the maximal grip force at all three resistances (data available for ipsilesional hand only, as explained in “Materials and methods”) (Fig. 3e). In Mk-LL subjected to PMd-r biopsy, in some contrast with the other two monkeys, the maximal grip force varied less systematically post-biopsy, as a significant decrease was limited to the left (ipsilesional) hand at resistance R3 (Fig. 3g).

As far as the maximal load force is concerned, the subjects were differentially affected (Fig. 3b, d, f, h). In Mk-MY (Fig. 3b), the maximal load force was not at all affected by the dlPFC biopsy, whereas, in Mk-LY, the dlPFC biopsy impacted on the maximal load force exerted by the right (contralesional) hand at all resistances (Fig. 3d) and by the left (ipsilesional) hand being not influenced. In Mk-MI, following dlPFC biopsy, there was a decrease of the load force at resistances R3 and R5 (Fig. 3f) for the ipsilesional hand. In Mk-LL (PMd-r lesion), a significant decrease of the maximal load force was observed for the left (ipsilesional) hand at resistances R3 and R5 and for the right (contralesional) hand at R5 only (Fig. 3h).

The global analysis of the grip force data revealed a significant decrease of the maximal grip force following the cortical biopsy (−1.20 ± 0.26, F1, 3455 = 185.50, *P* < 0.001). The same is true regarding the maximal load force (−0.29 ± 0.1, F1, 3455 = 73.27, *P* < 0.001). As expected the statistical analysis revealed a significant increase of both load and grip forces when incrementing the resistances (*P* < 0.0001). The interactions between the load force and the experimental phases had a significant effect on the load force (Type-III Anova: F_2_, 3455 = 3.06, *P* = 0.047) and on the grip force (Type-III Anova: F_2_, 3455 = 3.34, *P* = 0.036). The effects of the different resistances were further significantly lower during the post-biopsy phases for both the load and grip forces, with the exception of the effect of resistance 3 on the load force, which did not differ between the pre- and post-biopsy phases (Supplementary Table 1).

To sum up, following a kind of rostro-caudal biopsy gradient, Mk-MY with the most rostral biopsy exhibited post-biopsy, a decrease of the maximal grip force at all resistances and for both hands (Fig. 3a), without effect on the maximal load force. In Mk-LY, subjected to a somewhat more caudal (bigger) lesion in area 9, the maximal grip force was also affected (decrease) by the biopsy on both hands (with one exception, however, Fig. 3c), whereas some effect on the maximal load force amplitude appeared, but limited to the contralesional hand (Fig. 3d). In Mk-MI (ipsilesional hand only), the biopsy impacted on the maximal grip force at all resistances, though in the form of an increase, as well as on the load force (but decrease) at R3 and R5. Finally, in Mk-LL subjected to a lesion caudal to dlPFC, namely, in PMd-r, the effects appeared somewhat more lateralized, with the ipsilesional hand more affected (at three resistances for the maximal load and grip forces) than the contralesional hand (only the maximal load force at R5; Fig. 3h).

The data presented in Fig. 3 are a global comparison of the pre- versus post-biopsy periods, not showing the longitudinal changes of motor parameters from one daily session to the next. Longitudinal data for maximal grip force are presented in Supplementary Figs. 4 and 5, for two representative animals. Mk-MY, characterized by a significant decrease of maximal grip force post-biopsy (see Fig. 3a), without obvious recovery over a period of about 50 days, is illustrated in Supplementary Fig. 4. The maximal grip force was lower as of the first post-biopsy daily session, remaining on average lower and fairly stable during the whole post-biopsy period, although the intersession variability was largely comparable pre- versus post-biopsy. In contrast, Mk-LL is typical of an absence in most cases (hand; resistance) of difference of maximal grip force pre- versus post-biopsy (see Fig. 3g) and the longitudinal data are shown in Supplementary Fig. 5. Mk-LL exhibited a somewhat larger inter-sessions variability (than Mk-MY), and there was no evidence for a systematic change of maximal grip force during the first post-biopsy sessions which may have recovered in the subsequent sessions.

To address more precisely the predictive role of dlPFC for the control of grip forces, one may compare trials for which the monkey could not predict the grip force to apply, corresponding to the first trial at each resistance tested each day, with subsequent trials at each resistance for which the grip force to apply can be predicted. The analysis of first trials versus subsequent trials indeed showed some differences, in the sense that the maximal grip force applied by the monkey was generally higher in Mk-MY and Mk-MI and tented to be more variable (in all four monkeys) in the first trials than in the subsequent trials at the same resistance tested (data not shown). The systematic larger grip force in the first trials versus the subsequent trials was statistically significant at R0 (both hands) and at R3 (left hand) in Mk-MY as well as at R0 and R5 in Mk-MI. Although the grip force was generally more variable in the first trials than in the subsequent trials, this was only a trend, not statistically significant (*F* Test, *P* > 0.05).

## Discussion

**Fig. 4:**
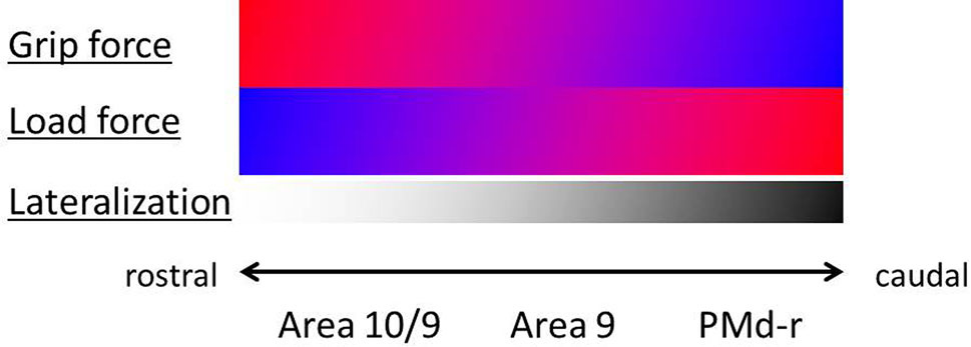
Schematic representation of a tentative rostro-caudal gradient of impact of unilateral lesions of the dlPFC (areas 10 and 9) or PMd-r on the control—prediction of grip and load forces and the lateralization of the effects on the load force, measured with the “reach and grasp drawer” task. The *red* to *blue* gradient represents the impact extent of the lesion according to the rostro-caudal position of the biopsy in dlPFC—PMd-r: in *red*, a strong impact and in *blue* a poor or absence of impact. In addition, the white to *black* gradient represents the lateralization of the effects on the load force according to the rostro-caudal position of the biopsy: in *white*, poor or no lateralized effect and in *black* stronger lateralized effect

In a general manner, the present study led to four main conclusions with respect to the role of the dlPFC in motor control:

1. As expected, lesions resulting from small biopsies in dlPFC and/or when located in areas 9/10 did not affect either the motor habit (spatiotemporal sequential strategies) or the motor performance (score) of manual dexterity in the “modified Brinkman board” task. The same conclusion holds true for a lesion located in PMd-r (area F7).
2. In sharp contrast, as revealed by the “reach and grasp drawer” task, significant modifications in the control of the maximal grip force were observed as a result of a lesion in areas 9/10, whereas the maximal load force was also affected, but to a lesser extent and only as a consequence of a more caudal lesion in area 9.
3. A lesion more caudal to dlPFC, in PMd-r (area F7), led to somewhat more lateralized hand (predominantly ipsilesional) and less systematic changes for both maximal grip and load forces.
4. Although limited to four cases, a rostro-caudal gradient of biopsy location appears to dictate the specific effects of the lesions in the “reach and grasp drawer” task (Fig. 4).

The above conclusion 1 valid for areas 9/10 and PMd-r is coherent with previous lesions in dlPFC targeted to area 46 (Kaeser et al. 2013) as far as the absence of effect on performance (score in the “modified Brinkman board” task) is concerned. On the other hand, a biopsy of 44 mm^3^ in area 46 led to a massive change of motor habit, whereas a medium size biopsy of 20 mm^3^ induced a moderate change of motor habit (Kaeser et al. 2013). This is in contrast with the present area 9/10 (7, 14, and 16 mm^3^) or PMd-c (7 mm^3^) lesions, which did not (or very little) impact on the “modified Brinkman board” data (neither score nor motor habit). At that step, it cannot be distinguished whether these differences related to the “modified Brinkman board” task between the present study, and the study of (Kaeser et al. 2013) is due to a difference in lesion size or to the location of the lesion, or to both.

In the context of cell therapy strategies, in particular regarding the ANCE approach, Kaeser et al. (2013) recommended to not exceed a dlPFC biopsy of 10 mm^3^ to limit deleterious effects on the motor habit and motor performance, as reflected by the “modified Brinkman board” task. The present results suggest that this upper limit of 10 mm^3^ may easily be raised up to 16 mm^3^ without a significant impact on motor habit and performance in the “modified Brinkman board” task. This observation has clinical implication, implying a reduced concern to apply the ANCE strategy to patients (stroke or Parkinson’s disease), as the present macaque monkey experiments suggest that unwanted motor consequences are unlikely, at least as far as motor habit and motor performance (in the “modified Brinkman board” task) are concerned in the context of a sequential grasping a small objects, which does not require the development of significant force levels. Therefore, in the case of strokes or edema when a surgical procedure is often required, the removed cortical material could be sorted and cryopreserved for possible subsequent ANCE production and treatment in the case of poor functional recovery (Brunet et al. 2002, 2003).

Unfortunately, due to the absence of significant spatiotemporal patterns’ modification of motor habit (Fig. 2), it was not possible to assess a potential hemispheric lateralization of such motor representations as previously suggested (Kaeser et al. 2013).

In contrast to the study of Kaeser et al. (2013) limited to the “modified and rotating Brinkman board” tasks (thus focused on grasping of small objects), the originality of the present study was to extend the consequences of dlPFC lesion to a substantially different motor control, involving the precise development of two forces, namely, grip force and load force. The above conclusions 2, 3, and 4 are consistent with the notion that dlPFC is involved in the precise control of predicted force, though more for the grip force than the load force. Indeed, as the trials on the “reach and grasp drawer” task were grouped according to the resistance level, after the first trial in each group of resistances (excluded from the analysis), the monkey knew the level of force required for each resistance and, therefore, could predict how much force was required in the subsequent trials. Indeed, the separate analysis of the first trials at each resistance tested daily showed that their grip force tented to be larger and more variable than at subsequent trials for which the forces to apply were predictable, based on the preceding first trial (working memory). The observed decrease of maximal grip force amplitude post-biopsy suggests that, after dlPFC or PMd-r lesion (to a lesser extent for the latter), there is a decrease in the margin of security to successfully grasp the drawer’s knob with enough force to prevent the fingers from slipping away from the knob. This phenomenon may favor an economy of energy, beneficial in the case of brain lesion, but at the cost to increase the risk of incorrect (unsuccessful) trials. Note, however, the case of Mk-MI (ipsilesional hand only) which also showed an impact of the biopsy on the maximal grip force, but in the other direction (increase post-biopsy), suggesting a possible loss in grip force control (to maximize effort as in the other monkeys). This divergent result in Mk-MI may be due to the position of the biopsy (more lateral than in Mk-LY), and/or a different recovery strategy from the lesion, and/or the asymmetry between both hands in the execution of the “reach and grasp drawer” task (as mentioned earlier).

The present data in the macaque monkey (except for Mk-MI) related to the prediction of grip force are well in line with the previous fMRI data reported for human subjects. Indeed, the role of prefrontal cortices (such as dlPFC), the cingulate motor area (CMA), as well as the ventral premotor area (PMv) has been widely reported to play a role in the control of grip force control in fMRI studies on human subjects (Rowe et al. 2000; Ehrsson et al. 2000, 2001; Kuhtz-Buschbeck et al. 2001, 2008). Furthermore, Vaillancourt and colleagues reported that the dlPFC and the anterior cingulate cortex (ACC) exhibited an increase of blood-oxygen-level-dependent (BOLD) signal when the task consisted of selecting the force amplitude (Vaillancourt et al. 2007). In addition, Wasson et al. (2010) demonstrated an activation of dlPFC, pre-SMA, and PMv in a task based on predictable grip force amplitude. In addition to cortical regions, the ventral thalamus, the cerebellum, and the anterior nuclei of the basal ganglia were activated suggesting a network encompassing all these regions to successfully execute grip force that require prediction. Moreover, our data are consistent with the previous reports on human subjects and strengthen the role of the dlPFC in the control (prediction) of the grip force (Ehrsson et al. 2000, 2001; Wasson et al. 2010; Neely et al. 2013) and its role in working memory (Pochon et al. 2001). In the human literature, dlPFC is activated predominantly on the side contralateral to the used hand, most often on the left side in the right-handed subjects. In the present study on macaques, alterations of grip and load (the latter to a lesser extent) forces control following unilateral dlPFC lesion were more variable, observed either for both hands, or the ipsilesional hand only and/or, less frequently, for the contralesional hand. The discrepancy with human data can be explained by the lack of strong manual lateralization at population level in *Macaca fascicularis*, although they may exhibit hand dominance/hand preference at individual level (Chatagny et al. 2013), but fairly equally balanced between the left and right hands. Moreover, the limited number of monkeys in the present study does not allow drawing firm conclusions about the laterality of dlPFC lesion effects at population level. Finally, human data are derived from functional imaging data, whereas the present monkey data are based on lesions, two approaches which may not yield fully comparable data. The control of dlPFC on the ipsilateral hand may involve projections to motor cortical areas (PMd, supplementary motor area, cingulate motor area) which are known to be bilaterally organized (e.g., Kermadi et al. 1998, 2000) or via the corpus callosum.

The case of monkey MK-LL, subjected to biopsy located not in the aimed dlPFC but in the adjacent PMd-r, illustrates that functional properties do not vary abruptly from one cortical area to the next, but rather exhibit a progressive transition. Indeed, the effect on maximal grip force was less present and less prominent than after lesion of area 9/10 (Fig. 3). Reciprocally, the effect on the maximal load force was somewhat more affected by the biopsy in PMd-r than in dlPFC (Fig. 3). Such a related load force control is consistent with previously reported roles of PMd in the control of proximal forelimb muscles (Freund and Hummelsheim 1985; Freund 1985; Fink et al. 1997).

The present data showing the role of dlPFC (mainly area 9/10) in the control of grip force (prediction) are consistent with the notion of a large neural circuit along the rostro-caudal axis, involving the prefrontal cortex (mostly for preparation and planning), the premotor cortex at large (mainly programming aspects), and primary motor cortex (principally involved in execution), responsible for the control of complex voluntary movements, with additional contributions from other brain structures (e.g., parietal cortex, basal ganglia, cerebellum, and brainstem). Along the rostro-caudal axis of this neural circuit, from prefrontal cortex to primary motor cortex, there is a progressive increase of lateralization, in the sense that caudally the motor control is predominantly focused on the contralateral forelimb, whereas it is more bilateral rostrally. Indeed, a dlPFC activation of both hemispheres during precision grip task has been reported in humans (Ehrsson et al. 2000). The same trend was found in the present data, comparing dlPFC and PMd (Fig. 4). The reduced lateralization of motor control in the rostral part of this neural circuit appeared clearly here in dlPFC (area 9/10) as the two animals with the most rostral biopsies exhibited significant effects on the control of grip force bilaterally (Fig. 3), which was not the case for the monkey subjected to a biopsy in PMd-r exhibiting a more lateralized effect.

To summarize, the present study provides new and complementary functional data regarding the role of dlPFC in motor habit representations and manual dexterity performances that support and extend the data and conclusions recently published by Kaeser and colleagues (2013). Moreover, due to its integrative conception, the “reach and grasp drawer” task developed by our laboratory allowed us to track subtle behavioral modifications in terms of grip and load forces’ control and their prediction. These data offer new interpretations related to lesions’ size and their precise location (e.g., area 46 versus areas 9/10) in dlPFC, as well as on the spatial functional organization of dlPFC along the rostro-caudal extent, with spread to the adjacent PMd-r area. However, due to the limited number of animals included in the present investigation, the interpretation of the data remains limited. Further investigations on a larger pool of monkeys are required to consolidate our hypotheses and conclusions at that step. Furthermore, the present data argue for the pertinence of the ANCE approach as cell therapy to treat brain lesion or neurodegeneration, based on biopsies targeted to dlPFC, although the size of the biopsy needs to be reduced as much as possible, to also avoid effects on the prediction and control of grip force levels. Although the biopsy in dlPFC led to statistically significant changes of grip force parameters and load force as well, but to a lesser extent, it is important to note that the monkeys were still able to perform the “reach and grasp drawer” task post-biopsy, based on modified motor parameters. It can thus be concluded that the ANCE approach can be recommended in the clinics, as it can be expected that the patient, such as the monkeys, will still be capable to perform manual grip actions, based on modified motor parameters, quickly adapted post-biopsy, especially if the size of biopsy will be proportionally smaller with respect to the total brain volume in humans than in monkeys.

## Electronic supplementary material

Below is the link to the electronic supplementary material.

**Supplementary Fig.** **1**. Same conventions as in Fig. 2A. The scores were compared pre- versus post-biopsy, based on the parametric Student unpaired *t* test, except for panel **D**. The results for each statistical comparison are indicated at the bottom right of each graph: n.s. = non-significant difference (p > 0.05); * is for p ≤ 0.05; ** is for p ≤ 0.01; *** is for P ≤ 0.001. Note that the two horizontal black lines (± 2 SDs) are missing in panel **D**, as the data do not follow a normal distribution (JPEG 761 kb)

**Supplementary Fig.** **2**. Same conventions as in Fig. 2B Note that for Mk-LL, the strategy analyses could not be performed due to its special task execution (see methods) (JPEG 5339 kb)

**Supplementary Fig.** **3**. Same conventions as in Fig. 2D. Note that for Mk-LL, the strategy analyses could not be performed due to its special task execution. The indexes were compared pre- versus post-biopsy, based on the non-parametric Mann and Whitney test or the parametric Student unpaired *t* test. The results for each statistical comparison are indicated on the bottom right of each graph: n.s. = non-significant difference (p > 0.05); ** is for p ≤ 0.01 (JPEG 684 kb)

**Supplementary Fig.** **4**. Graphs showing the longitudinal distribution of the maximal grip forces of Mk-MY, from one daily behavioral session to the next in both the pre-biopsy and post-biopsy periods. Data points aligned in one column vertically correspond to the five trials usually considered in each session (several trials were discarded in each session based on strict inclusion criteria). Each dot represents one trial. The biopsy took place at day 0, represented by the vertical red line. The panels A, C, and E show the left hand (LH), whereas the right hand (RH) is represented in panels B, D, and F. The three resistances (R0, R3, and R5) are ordered from the top to the bottom: top row: R0, middle row: R3 and bottom row: R5. The maximal grip force values were compared between pre- versus post-biopsy periods, based on the non-parametric Mann and Whitney test or the parametric Student unpaired *t* test. The results for each statistical comparison are indicated at the bottom right of each graph: *** is for P ≤ 0.001 (JPEG 728 kb)

**Supplementary Fig.** **5**. Graphs showing the longitudinal distribution of the maximal grip forces of Mk-LL, following the same conventions as in Supplementary Fig. 4. The results for each statistical comparison are indicated at the bottom right of each graph: n.s. = non-significant difference (p > 0.05); ** is for p ≤ 0.01 (JPEG 586 kb)

**Supplemantary Table** **1**: Summary of the results of the linear mixed model comparison performed in R (see methods and results). Significance threshold = 0.05. DF = degree of freedom (PDF 29 kb)
