# Supplementary figures and images for: Effects of dorsolateral prefrontal cortex lesion on motor habit and performance assessed with manual grasping and control of force in macaque monkeys
Source: Brain Struct Funct. 2016 Jul 9;222(3):1193–206. doi: 10.1007/s00429-016-1268-z (PMC5368204; doi:10.1007/s00429-016-1268-z)

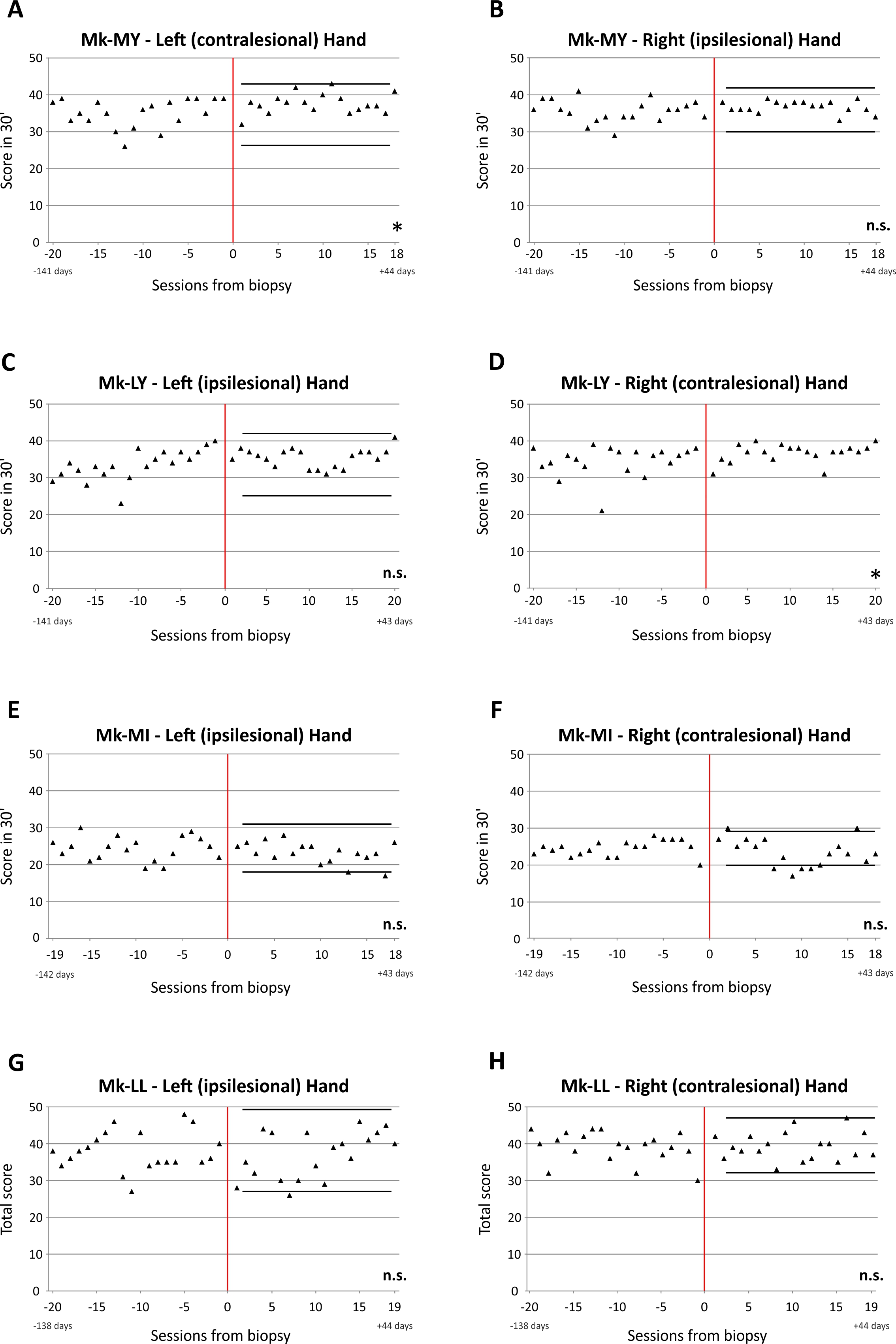

Supplement: Supplementary file 1 — Supplementary Fig. 1. Same conventions as in Fig. 2A. The scores were compared pre- versus post-biopsy, based on the parametric Student unpaired t test, except for panel D. The results for each statistical comparison are indicated at the bottom right of each graph: n.s. = non-significant difference (p > 0.05); * is for p ≤ 0.05; ** is for p ≤ 0.01; *** is for P ≤ 0.001. Note that the two horizontal black lines (± 2 SDs) are missing in panel D, as the data do not follow a normal distribution (JPEG 761 kb) [file 429_2016_1268_MOESM1_ESM.jpg]

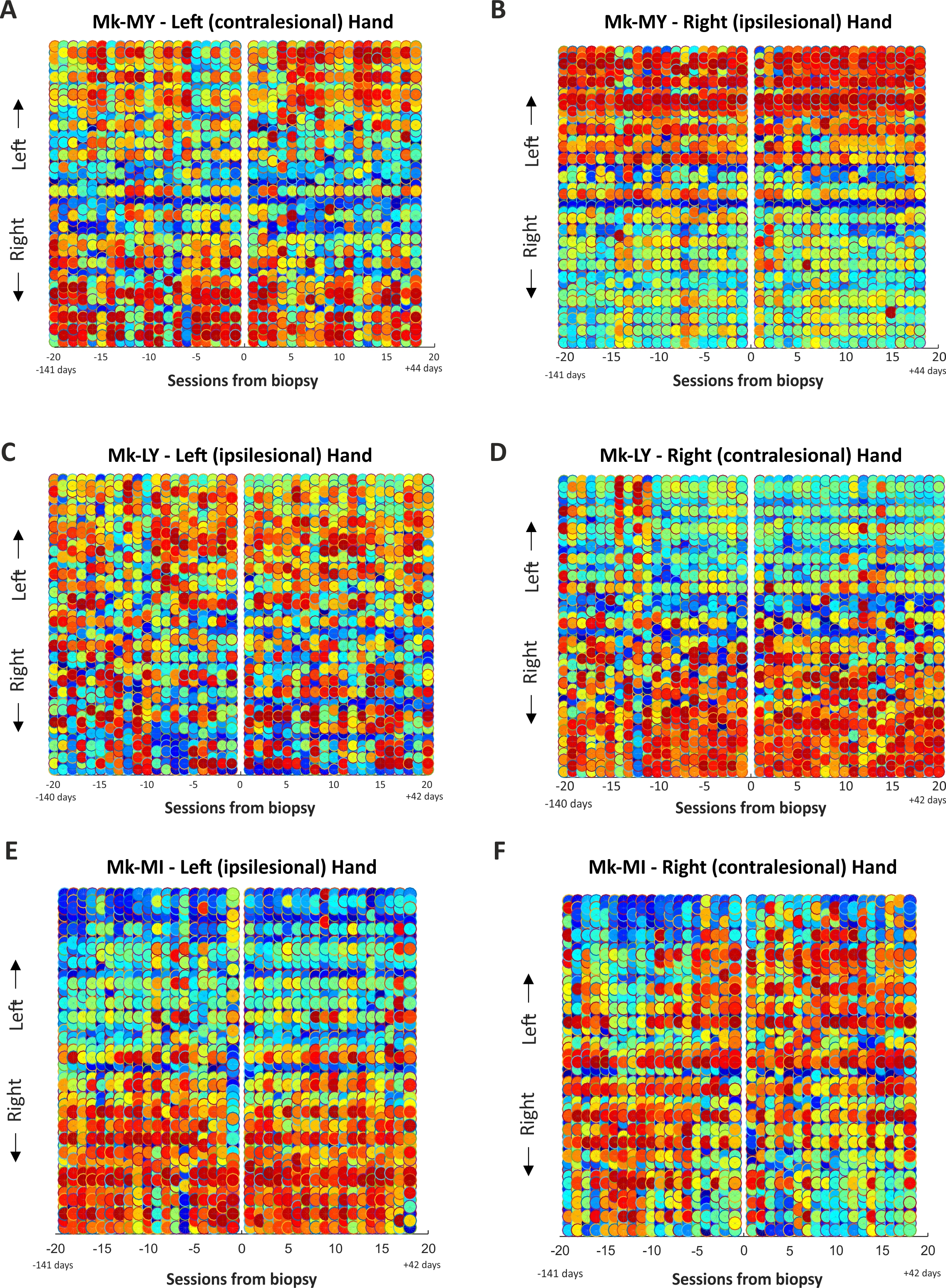

Supplement: Supplementary file 2 — Supplementary Fig. 2. Same conventions as in Fig. 2B Note that for Mk-LL, the strategy analyses could not be performed due to its special task execution (see methods) (JPEG 5339 kb) [file 429_2016_1268_MOESM2_ESM.jpg]

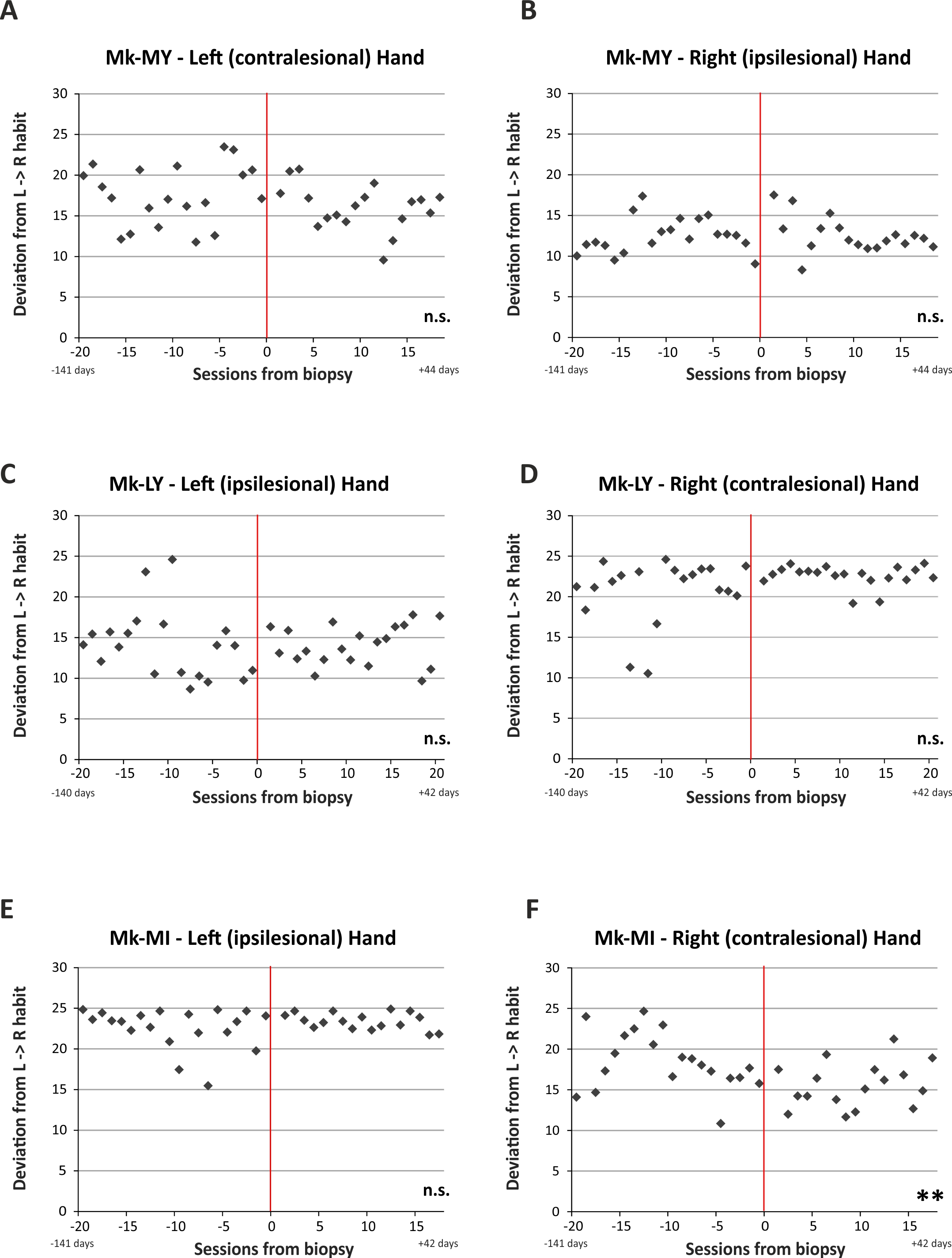

Supplement: Supplementary file 3 — Supplementary Fig. 3. Same conventions as in Fig. 2D. Note that for Mk-LL, the strategy analyses could not be performed due to its special task execution. The indexes were compared pre- versus post-biopsy, based on the non-parametric Mann and Whitney test or the parametric Student unpaired t test. The results for each statistical comparison are indicated on the bottom right of each graph: n.s. = non-significant difference (p > 0.05); ** is for p ≤ 0.01 (JPEG 684 kb) [file 429_2016_1268_MOESM3_ESM.jpg]

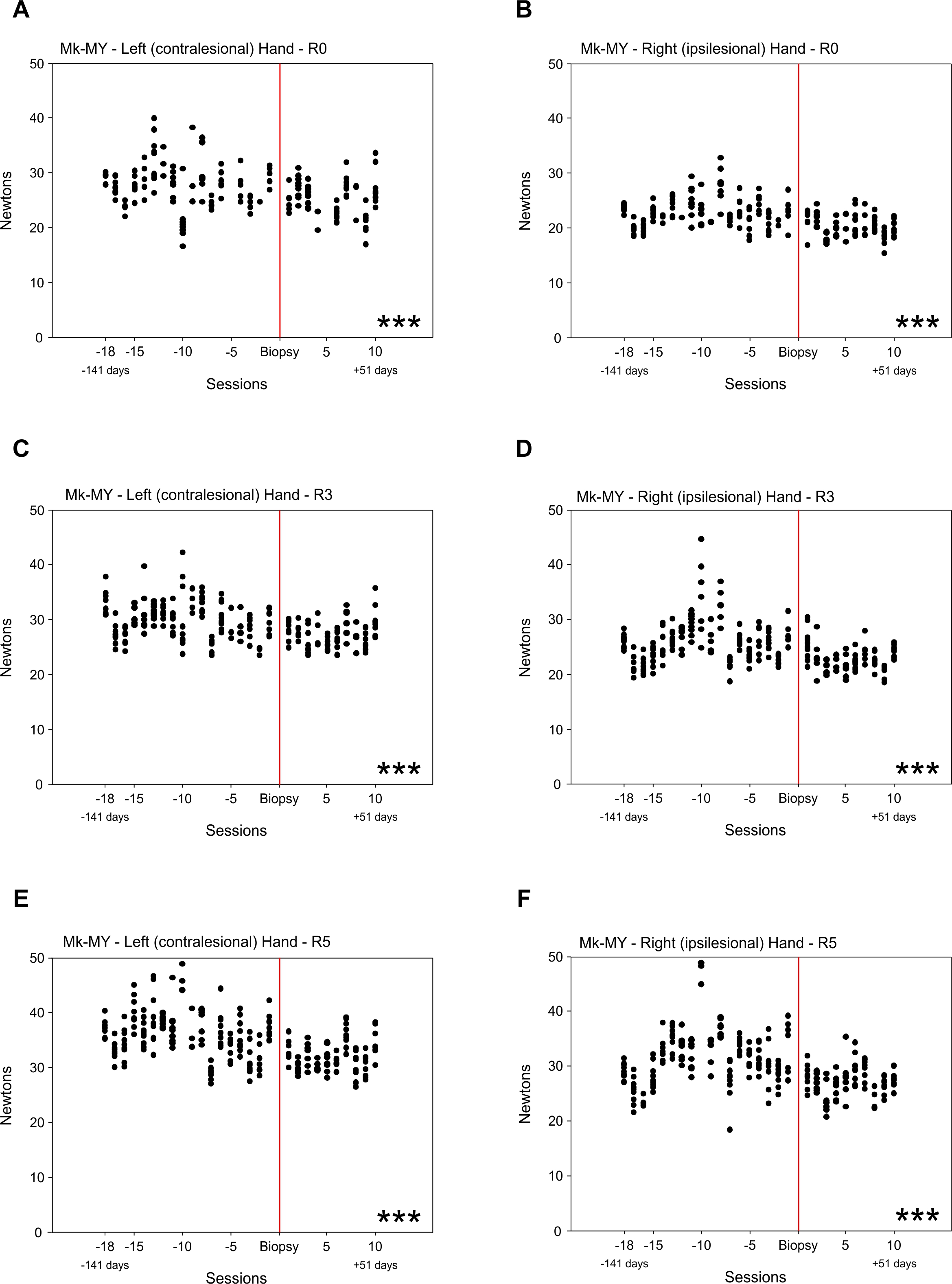

Supplement: Supplementary file 4 — Supplementary Fig. 4. Graphs showing the longitudinal distribution of the maximal grip forces of Mk-MY, from one daily behavioral session to the next in both the pre-biopsy and post-biopsy periods. Data points aligned in one column vertically correspond to the five trials usually considered in each session (several trials were discarded in each session based on strict inclusion criteria). Each dot represents one trial. The biopsy took place at day 0, represented by the vertical red line. The panels A, C, and E show the left hand (LH), whereas the right hand (RH) is represented in panels B, D, and F. The three resistances (R0, R3, and R5) are ordered from the top to the bottom: top row: R0, middle row: R3 and bottom row: R5. The maximal grip force values were compared between pre- versus post-biopsy periods, based on the non-parametric Mann and Whitney test or the parametric Student unpaired t test. The results for each statistical comparison are indicated at the bottom right of each graph: *** is for P ≤ 0.001 (JPEG 728 kb) [file 429_2016_1268_MOESM4_ESM.jpg]

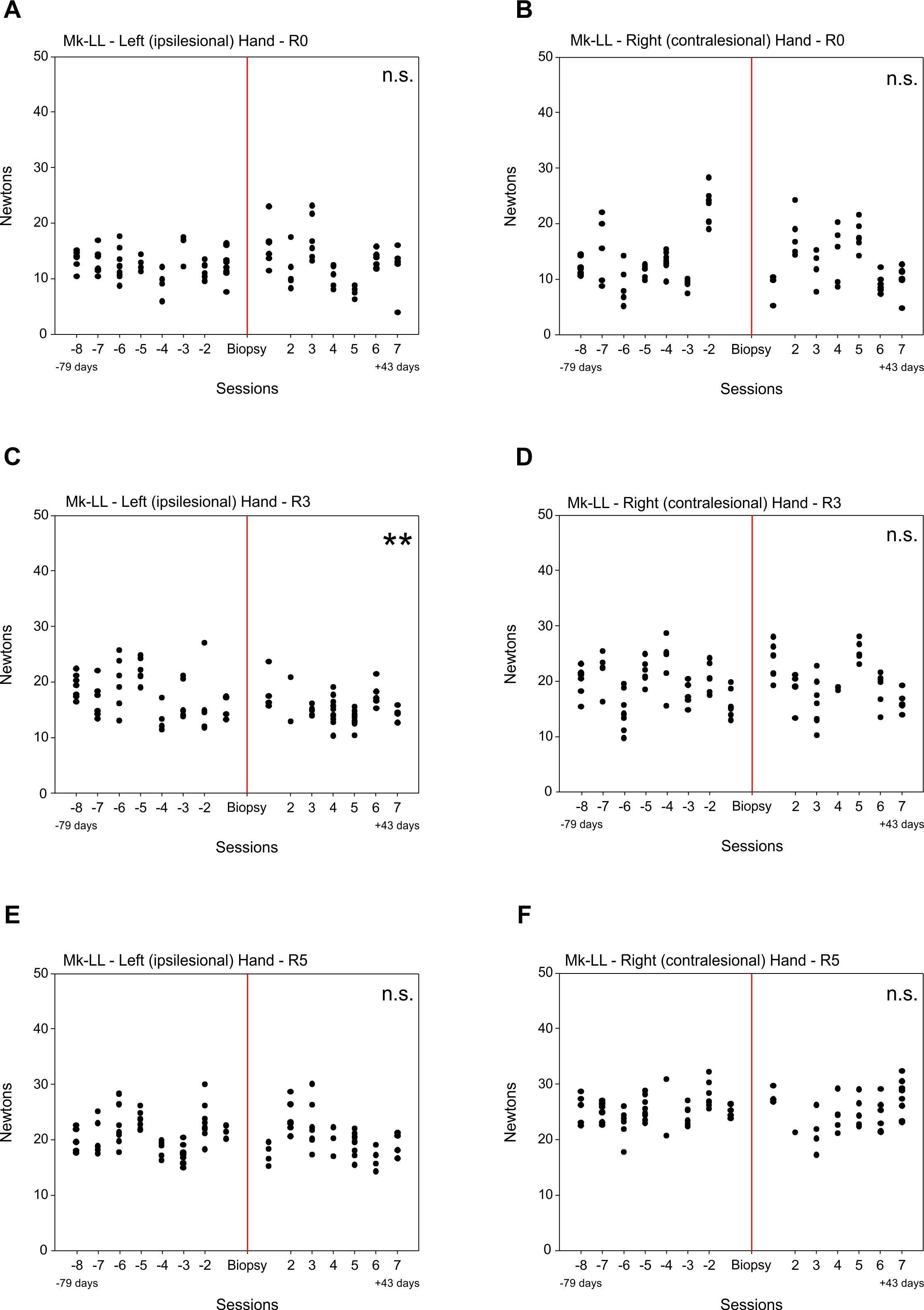

Supplement: Supplementary file 5 — Supplementary Fig. 5. Graphs showing the longitudinal distribution of the maximal grip forces of Mk-LL, following the same conventions as in Supplementary Fig. 4. The results for each statistical comparison are indicated at the bottom right of each graph: n.s. = non-significant difference (p > 0.05); ** is for p ≤ 0.01 (JPEG 586 kb) [file 429_2016_1268_MOESM5_ESM.jpg]
