# Supplementary material for: Effects of dorsolateral prefrontal cortex lesion on motor habit and performance assessed with manual grasping and control of force in macaque monkeys
Source: Brain Struct Funct. 2016 Jul 9;222(3):1193–206. doi: 10.1007/s00429-016-1268-z (PMC5368204; doi:10.1007/s00429-016-1268-z)
Supplement: Supplementary file 6 — Supplemantary Table 1: Summary of the results of the linear mixed model comparison performed in R (see methods and results). Significance threshold = 0.05. DF = degree of freedom (PDF 29 kb) [file 429_2016_1268_MOESM6_ESM.pdf]

Max grip force

	Value	Std.Error	DF	t-value	p-value
Intercept	20.64	2.99	3455	6.91	<0.0001
Resistance 3	3.49	0.21	3455	16.28	<0.0001
Resistance 5	8.29	0.21	3455	39.24	<0.0001
Biopsy	-1.20	0.26	3455	-4.61	<0.0001
**Interactions**					
Resistance 3 : biopsy	-0.69	0.35	3455	-1.96	0.05
Resistance 5 : biopsy	-0.87	0.35	3455	-2.49	0.01

Max load force

	Value	Std.Error	DF	t-value	p-value
Intercept	5.38	0.44	3455	12.25	<0.0001
Resistance 3	2.36	0.08	3455	28.30	<0.0001
Resistance 5	5.41	0.08	3455	66.00	<0.0001
Biopsy	-0.29	0.10	3455	-2.91	<0.001
**Interactions**					
Resistance 3 : biopsy	-0.01	0.14	3455	-0.04	0.97
Resistance 5 : biopsy	-0.28	0.14	3455	-2.08	0.04
